# Genome-wide analysis of *HSP70* gene superfamily in kelp (*Saccharina japonica*): identification, characterization, and heat stress-responsive expression profiles

**DOI:** 10.7717/peerj.21451

**Published:** 2026-06-26

**Authors:** Zhongyuan Lin, Wentai Mao, Yijuan Han, Huibin Xu, Ximin Qiu, Yanfei Liu, Weiqi Tang, Hongmei Lin, Yongji Huang, Wenshan Wang, Wenwei Lin, Jianjun Lu, Songbiao Chen

**Affiliations:** 1 Fujian Provincial Universities Engineering Research Center of Marine Biology and Drugs, College of Geography and Oceanography, Minjiang University, Fuzhou, China; 2 Fujian Key Laboratory on Conservation and Sustainable Utilization of Marine Biodiversity, College of Geography and Oceanography, Minjiang University, Fuzhou, China; 3 Marine Biotechnology Center, Fuzhou Institute of Oceanography, Fuzhou, China; 4 Fuzhou Technological Innovation Center of Seawater Planting Industry, Minjiang University, Fuzhou, China; 5 College of Life Sciences, Fujian Agriculture and Forestry University, Fuzhou, China; 6 Fujian Fisheries Technology Extension Center, Fuzhou, China

**Keywords:** *Saccharina japonica*, HSP70, Expression pattern, Heat stress

## Abstract

With the ongoing global climate change, high temperatures severely compromise the cultivation of large seaweeds. *Saccharina japonica*, one of the most economically important alga species, is a cold-temperate seaweed particularly sensitive to heat stress. However, the regulatory mechanisms underlying the response of *S. japonica* to heat stress remain unclear. Heat shock proteins (HSPs) play a crucial role in protecting plants against various abiotic stresses. Although individual members of the HSP70 family have been analyzed in previous studies, a comprehensive analysis of the *HSP70* gene family in *S. japonica* has not yet been conducted. In this study, we identified fourteen putative *HSP70* genes in *S. japonica*, of which four belong to the HSP110 subfamily and ten to the DnaK subfamily. Furthermore, we characterized the expression patterns of the *SjHSP70* genes. Among them, ten *HSP70s* were up-regulated under heat stress. Subcellular localization analysis further revealed that SjHSP70-03, SjHSP70-06, and SjHSP70-13 were mainly localized in the nucleus/cytoplasm, whereas SjHSP70-11 was also detected in the nucleus and cytoplasm but predominantly in an undetermined compartment. These findings provide valuable insights that will facilitate future studies on the function of HSP70s in *S. japonica.*

## Introduction

As global climate change leads to rising temperatures, heat stress has emerged as a critical factor affecting the growth and yield of seaweeds. To improve their survival under changing temperature conditions, seaweeds have developed mechanisms that regulate the acclimation of stress-related genes (Vierling, 2003). Among these mechanisms, heat shock proteins (HSPs) play crucial roles in stress responses (Feder & Hofmann, 1999). HSPs are essential for cellular protection against abiotic stress, functioning as chaperone proteins that stabilize proteins, prevent aggregation, and facilitate proper folding (Jacob, Hirt & Bendahmane, 2017; Wang et al., 2004). Based on their molecular weights, HSPs were classified into five major families: HSP100, HSP90, HSP70, HSP60, and small heat shock proteins (sHSPs) (Al-Whaibi, 2011). Notably, HSP70s are a highly conserved family of HSPs and have been shown to play a significant role in various stress responses, contributing notably to developmental processes and defense mechanisms (Usman et al., 2017).

The conserved architecture of HSP70s comprises a nucleotide binding domain (NBD), a substrate binding domain (SBD), and a C-terminal domain (Usman et al., 2017). Based on predicted subcellular localization, HSP70s are classified into four subgroups in plants: cytosolic (C) HSP70s, endoplasmic reticulum (ER) HSP70s, mitochondrial (Mit) HSP70s, and chloroplast (CP) HSP70s (Sung, Vierling & Guy, 2001). Studies have demonstrated that HSP70s play a crucial role in responding to heat stress in many organisms. For example, the HSP70-HSP40 chaperone complex is critical for heat stress responses in *Arabidopsis* (Wang et al., 2021). Overexpression of *HSP70s* from various plant species, such as *Macrotyloma uniflorum* (Masand & Yadav, 2016), *Paeonia lactiflora* (Zhao et al., 2019), and *Zostera japonica* (Chen & Qiu, 2022), in *A. thaliana* has been shown to improve high-temperature tolerance. Similarly, HSP70s also play a critical role in thermal tolerance responses in algae. For instance, HSP70 has been identified as a potential biomarker of environmental stress, including thermal stress, in *Enteromorpha intestinalis* (Lewis, Donkin & Depledge, 2001). Overexpression of the mitochondrial *PsHSP70b* in *Chlamydomonas* has been shown to enhance heat resistance (Park et al., 2012). The expression of *HSP70s* has been demonstrated to be dramatically up-regulated by temperature in green algae (Deng et al., 2015b), dinoflagellates (Deng et al., 2015a), red algae (Huang et al., 2024; Mikami & Khoa, 2023), and brown algae (Fu et al., 2009; Rathor et al., 2023).

*S. japonica* is an economically important seaweed, with the largest scale of cultivation and production globally (Liu et al., 2024). The yield and quality of cultivated *S. japonica* have been affected by climate change, particularly by the frequency of heatwaves (Gao, Endo & Agatsuma, 2015; Hu et al., 2024). The optimum temperature for *S. japonica* is 10–15 °C, and the critical temperature at which the alga experiences heat stress exceeds 24 °C (Borlongan et al., 2020). In production practice, 18–20 °C is the threshold for transferring young sporophytes to open-sea cultivation. Therefore, testing at 25 °C can help assess the feasibility of early transfer, which is relevant to commercial farming (Ling et al., 2016; Lu et al., 2023).

Previous studies have demonstrated that *HSP70s* were induced in *S. japonica* sporophytes in response to heat stress, suggesting that HSP70s may play important roles in heat stress responses (Fu et al., 2009; Lin et al., 2025), similar to plants or other algae. However, a genome-wide characterization of the HSP70 family in *S. japonica* remains unexplored. In this study, 14 *HSP70* genes were identified in the genome of *S. japonica*. Comprehensive analyses of these *SjHSP70* genes were carried out, including phylogenetic relationships, chromosomal distribution, gene structures, and conserved motifs. Moreover, expression profiles of the *SjHSP70* genes were examined using existing RNA-seq datasets to assess their potential roles across various tissues during different developmental stages and in response to abiotic stresses. These findings contribute to a better understanding of the functional roles of *HSP70* genes in *S. japonica* and lay the groundwork for future research on stress response mechanisms in this species.

## Materials and Methods

### Identification of *SjHSP70* family genes

Nucleic acid and protein sequences of *S. japonica* were retrieved from the Online Resource for Community Annotation of Eukaryotes (ORCAE; https://bioinformatics.psb.ugent.be/orcae/overview/Sacja) and GCA_048937375.1 (Guo et al., 2025) (Table S1). The Hidden Markov Model profiles of the HSP70 domain (PF00012) obtained from the Pfam database (http://pfam.sanger.ac.uk/) were used to identify the HSP70 proteins in the *S. japonica* dataset using simple HMMER search software in TBtools. Those with an E-value <0.001 were selected. The identified *SjHSP70* genes were re-analyzed to confirm the existence of the HSP70 domain by InterPro databases (http://www.ebi.ac.uk/interpro/). The known HSP70 protein sequences from *Ectocarpus siliculosus* were retrieved from the Online Resource for Community Annotation of Eukaryotes (https://bioinformatics.psb.ugent.be/orcae). The identified HSP70 protein sequences of *Chlamydomonas reinhardtii* and *A. thaliana* were downloaded from the Phytozome database. HSP70 protein sequences of *Undaria pinnatifida*, *Alaria crispa*, *Neopyropia yezoensis*, and *Pyropia haitanensis* were downloaded from the National Center for Biotechnology Information (NCBI) database.

The chromosomal locations, genomic sequences, full coding sequences, protein sequences, and 2.0 kb upstream regions from the translation start codon were downloaded from ORCAE or GCA_048937375.1. Molecular weight (Da) and isoelectric point (pI) were calculated for each gene using the ProtParam tool in ExPASy (https://www.expasy.org/).

### Chromosome mapping and phylogenetic analysis of HSP70 family members

The chromosomal distribution of the predicted *SjHSP70* genes was mapped using Mapchart software (Guo et al., 2025). To construct phylogenetic trees, amino acid sequences of *S. japonica* and seven other species (*E. siliculosus*, *U. pinnatifida*, *A. crispa*, *N. yezoensis*, *P. haitanensis*, *C. reinhardtii*, and *A. thaliana*) were used. Multiple sequences alignments were performed using ClustalW in MEGA 11.0 with default parameters. Phylogenetic trees were then generated using the Maximum Likelihood (ML) algorithm and the Jones Taylor Thornton (JTT) model with default parameters. Confidence values were obtained with bootstrapping with 1,000 replications. The tree was further visualized using the Interactive Tree Of Life (iTOL) platform.

### Protein and DNA sequence analyses

Conserved motifs of HSP70 protein sequences were identified using the online MEME tool (https://meme-suite.org/meme/tools/meme) (Table S2). The exon-intron gene structures of the HSP70 members were analyzed by aligning the CDS with their corresponding genomic DNA sequences (excluding UTRs), and the results were visualized using the Gene Structure Display Server tool (GSDS: http://gsds.gao-lab.org/).

### Analysis of the cis-regulatory elements in the promoter

To identify cis-regulatory elements, the 2.0 kb upstream sequences from the start codon related to each *SjHSP70* gene were analyzed using PlantCARE software (https://bioinformatics.psb.ugent.be/webtools/plantcare/html/). The expression profiles of cis-regulatory elements were constructed using the pheatmap software.

### Expression analysis of *HSP70* genes

To investigate the expression profile of *SjHSP70* genes during different developmental stages and under various treatments (including copper ion stress, salt stress, and heat stress), we retrieved publicly available RNA-seq from NCBI and the China National Center for Bioinformation (CNCB). Specifically, the datasets for copper ion stress (Cu^2+^, at 0, 10, 100, and 200 μg·L^−1^ treatment; BioProject accession: PRJNA387211) and heat stress (25 °C, at 0, 3, and 6 h after treatment; BioProject accession: PRJNA949272); the dataset for different tissue types (rhizoids, stipes, blade tips, blade pleats, blade bases, and blade fascia for *Sja* sporophytes under normal condition; BioProject accession: PRJCA000815) and hyposaline stress (control salinity: 30‰, hyposaline: 12‰; BioProject accession: PRJCA000815) was obtained from CNCB, as described in previous studies (Chi et al., 2020; Lin et al., 2025; Zhang et al., 2019). The data of RNA-seq were analyzed using hisat2 (Kim et al., 2019). Gene expression levels were determined as transcripts per kilobase million (TPM) values. Differentially expressed genes were identified using the thresholds |log_2_FC| > 1, *p* value < 0.05. The Heatmap was constructed using R software and based on the log_10_ (TPM+1).

### Sample collection and quantitative real-time PCR (qRT-PCR) validation

The healthy young sporophytes (20–30 cm in length) of *S. japonica* “Huangguan No.1” were cultured at 25 °C for 0, 3, and 6 h, respectively. Samples were then harvested, immediately frozen in liquid nitrogen, and stored at –80 °C until RNA extraction. Each group consisted of three biological replicates.

Total RNAs were extracted using Trizol reagent (Invitrogen, Carlsbad, CA, USA) and treated with RNase-free DNase I (Takara, Beijing, China) to remove genomic DNA contamination. First-strand cDNA was synthesized from 1 μg of RNA using the HiScript II 1st Strand cDNA Synthesis Kit (Vazyme, Nanjing, China). Each cDNA sample was diluted 10-fold in sterile water prior to qRT-PCR analysis. The qRT-PCR reactions were performed on a CFX Connect Real-Time System (BIO-RAD, Hercules, CA, USA) using the SYBR Mix (Vazyme, Nanjing, China). Three replications were conducted for each sample. The housekeeping gene *β-actin* (*SJ13834*) was used as an internal control. Relative expression levels were calculated using the 2^−ΔΔCT^ method (Livak & Schmittgen, 2001). Primer sequences are listed in Table S3.

### Subcellular localization analysis of SjHSP70s

The coding sequences of four SjHSP70s were cloned and fused to GFP in the pRTVcGFP vector, respectively. The *GFP* alone construct and each *SjHSP70-GFP* construct were co-transfected with the nucleus marker construct *OsbZIP72-mCherry* into rice protoplasts *via* a PEG-mediated transfection method, as previously described (Chen et al., 2006).

### Statistical analysis

The data are reported as the mean ± standard deviation from at least three replicates. Statistical analyses were performed using Microsoft Office Excel 2016 and GraphPad Prism 9.00 software. Significance levels were determined by conducting a one-way analysis of variance (ANOVA). All bar charts were created utilizing GraphPad Prism 9.00.

## Results

### Genome-wide identification and chromosomal distribution of *SjHSP70* genes in the *S. japonica* genome

To identify *HSP70* genes in the *S. japonica* genome, a BLAST search was performed using the conserved HSP70 domain (Pfam: PF00012) as the query. The candidate sequences were further validated using HMMER based on TBtools. As a result, a total of 14 *SjHSP70* genes were identified (Tables 1, S1). These genes were named *SjHSP70-01* to *SjHSP70-14* based on their chromosomal positions. The predicted proteins ranged from 469 (SjHSP70-03) to 1,130 (SjHSP70-04) amino acids in length, with corresponding molecular weights varying from 49.91 to 116.67 kDa. The theoretical isoelectric points (pIs) of most predicted SjHSP70s were below six, except for SjHSP70-04 and SjHSP70-09 (Table 1).

**Table 1:** List of 14 *HSP70* genes identified in *S. japonica*.

Gene name	Guo et al. (2025)		Ye et al. (2015)		CDS length (bp)	Protein length (aa)	pI	MW (Da)	Instability index
	Gene ID	Location coordinates (5′–3′)	Gene ID	Location coordinates (5′–3′)					
SjHSP70-1	Sja001084	Chr3:1,907,059–1,924,023	SJ19807	chr10:1,236,720–1,247,128	2,211	737	5.51	79,572.01	46.62
SjHSP70-2	Sja001189	Chr3:4,854,345–4,861,794	SJ00203	chr10:3,619,811–3,627,260	1,974	658	4.73	72,402.75	34.69
SjHSP70-3	Sja003276	Chr6:10,545,960–10,554,755	SJ16867	chr11:7,030,926–7,039,694	1,407	469	5.33	49,905.5	41.66
SjHSP70-4	Sja003999	Chr7:16,174,426–16,200,941	SJ07114	chr8:10,570,966–10,594,816	3,390	1,130	6	116,671.33	50.16
SjHSP70-5	Sja005508	Chr11:2,420,781–2,438,402	SJ11938	chr9:11,972,288–11,989,398	2,586	862	5	92,502.38	30.51
SjHSP70-6	Sja005969	Chr11:14,307,993–14,319,324	SJ06971	chr6:6,352,368–6,361,573	1,956	652	5.07	69,485.31	32.88
SjHSP70-7	Sja006503	Chr12:14,534,806–14,563,633	SJ07715	chr15:11,261,677–11,285,799	2,145	715	4.7	74,902.73	42.48
SjHSP70-8	Sja007040	Chr14:4,646,512–4,684,211	SJ07797	chr0:51,087,343–51,110,146	2,526	842	5.05	90,090.07	36.97
SjHSP70-9	Sja008986	Chr18:6,793,784–6,818,382	SJ09592	chr18:4,145,224–4,170,781	2,238	746	7.15	77,083.91	39.36
SjHSP70-10	Sja012376	Chr26:8,356,028–8,376,858	SJ15841	chr13:3,240,159–3,259,333	2,172	724	4.96	77,722.16	27.72
SjHSP70-11	Sja012625	Chr27:4,499,930–4,533,467	SJ21930	chr27:5,986,364–6,005,462	2,082	694	5.12	73,945.86	40.14
SjHSP70-12	Sja014729	Chr32:1,253,216–1,272,677	SJ03579	chr0:182,637,878–182,647,937	2,013	671	4.94	73,312.47	37.97
SjHSP70-13	Sja014731	Chr32:1,280,213–1,285,456	SJ09756	chr30:1,613,497–1,618,911	2,013	671	5.1	73,583.04	39.29
SjHSP70-14	Sja014732	Chr32:1,286,555–1,290,997	SJ09757	chr30:1,607,892–1,612,398	1,974	658	5.01	72,120.48	39.28

**Figure 1:**
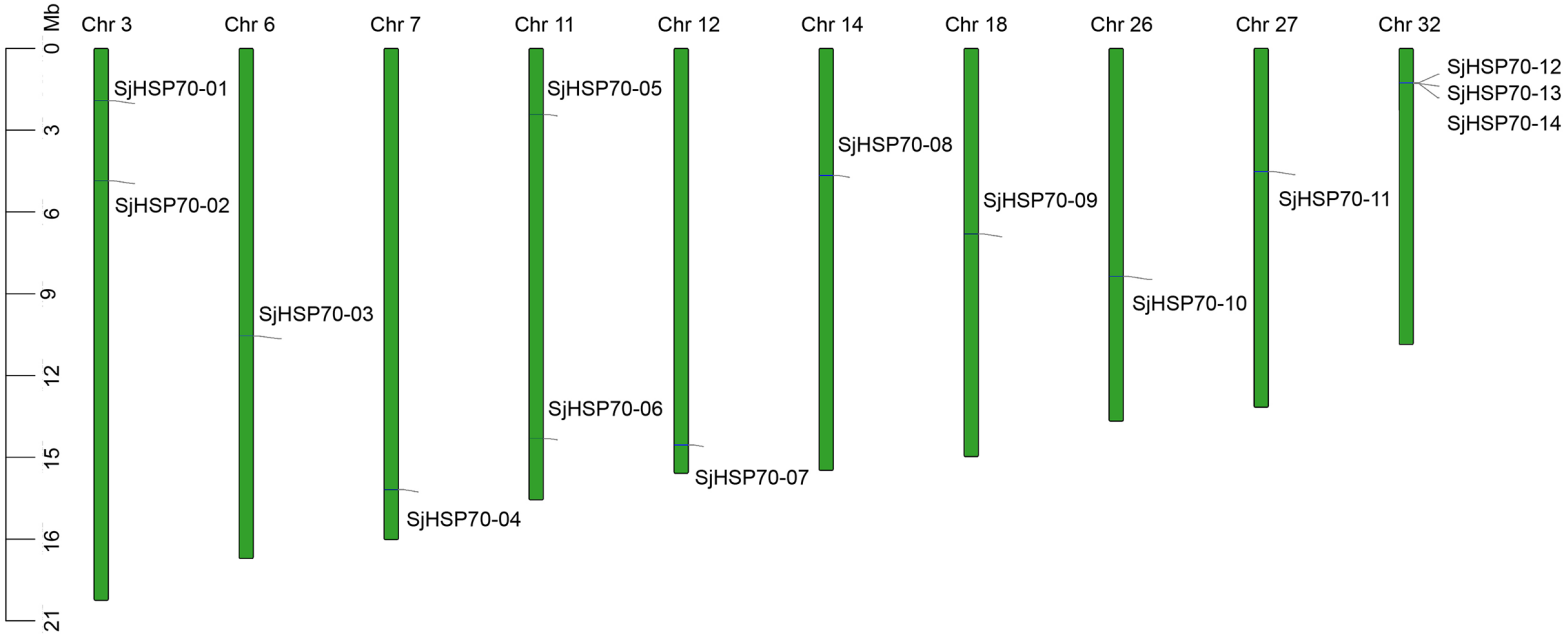
Distribution of predicted *SjHSP70* genes in the *S. japonica* genome. Note: Chr, Chromosome.

The 14 *SjHSP70* genes were unevenly distributed across 10 chromosomes (Fig. 1). Chromosomes Chr6, Chr7, Chr12, Chr14, Chr18, Chr26, and Chr27 each harbored a single *SjHSP70*, while Chr3 and Chr11 each contained two *SjHSP70s*, and Chr32 contained three *SjHSP70s*.

### Phylogenetic analysis of SjHSP70 proteins

**Figure 2:**
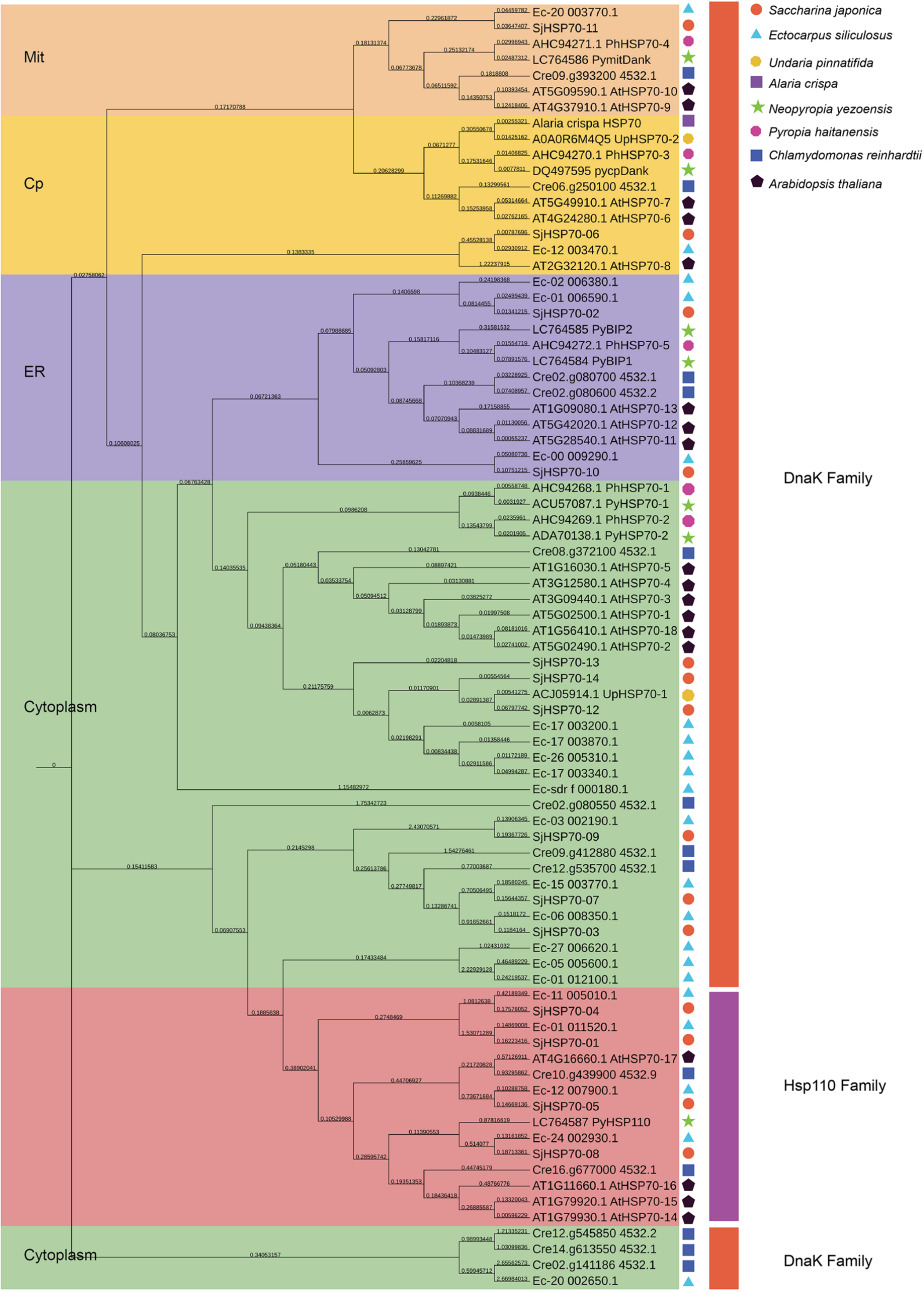
Phylogenetic analysis of HSP70 proteins from *S*. *japonica* and other species. The analysis included 38 brown algal HSP70s (14 from *S. japonica*, 21 from *E. siliculosus*, 2 from *U. pinnatifida*, and 1 from *A. crispa*), 11 red algal HSP70s (7 from *N. yezoensis* and 4 from *P. haitanensis*), 13 green algal HSP70s from *C. reinhardtii*, and 18 HSP70s from *A. thaliana*. HSP70 protein sequences were aligned by ClustalW in MEGA 11.0 with default parameters. Maximum Likelihood (ML) method was used to construct the phylogenetic tree. The numbers beside all branches represent branch lengths. CP, chloroplast; ER, endoplasmic reticulum; Mit, mitochondrion.

The full-length amino acid sequences of HSP70 family members from *S. japonica, E. siliculosus*, *U. pinnatifida*, *A. crispa*, *N. yezoensis*, *P. haitanensis*, *C. reinhardtii*, and *A. thaliana* were aligned using ClustalW, and a phylogenetic tree was constructed. As shown in Fig. 2, the HSP70 protein superfamily was grouped into two subfamilies: HSP110 and DnaK. Four SjHSP70s were assigned to the HSP110 subfamily, and 10 to the DnaK subfamily (Fig. 1). The DnaK subfamily was further divided into four clades (Fig. 1). In particular, one SjHSP70s clustered in the Mit clade, one in the CP clade, two in the ER clade, and six in the Cytoplasm clade. The SjHSP70s showed higher homology to those from other algal species than to those from *A. thaliana*, indicating a closer evolutionary relationship among algae.

### Gene structures of *SjHSP70* genes and conserved motifs of SjHSP70 proteins

**Figure 3:**
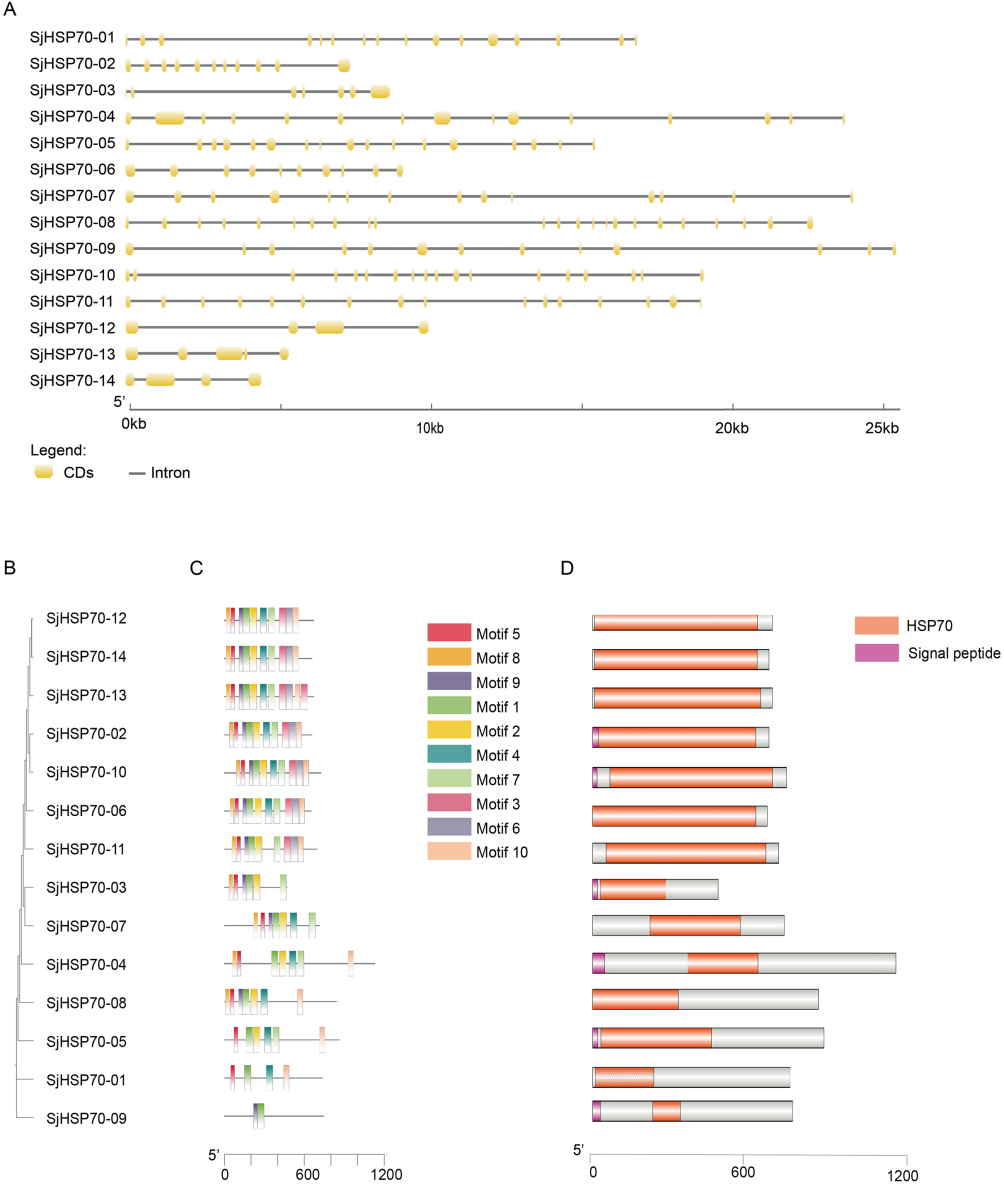
Exon/intron structures, phylogenetic relationships, conserved motifs, and domains of HSP70s in *S*. *japonica*. (A) Exon/intron structures of *SjHSP70s*. Exons are indicated by yellow boxes, and introns by black lines. (B) Phylogenetic trees of SjHSP70s. The unrooted tree was constructed with MEGA 11.0 using full-length amino acid sequences of the 14 SjHSP70s by the Maximum Likelihood (ML) method. (C) Conserved motifs in SjHSP70s. (D) HSP70 domains in SjHSP70s. The lengths of exons, introns, motifs, and domains can be estimated using the scale at the bottom.

To explore the structural organization of *SjHSP70* genes, we examined the exon-intron structure of each member (Fig. 3A). Intron numbers varied from three (in *SjHSP70-12* and *SjHSP70-14*) to 22 (in *SjHSP70-08*), with considerable variability in intron length and sequence.

To characterize the structural features of SjHSP70 proteins, we analyzed their conserved motifs and domains (Figs. 3B–3D). MEME analysis identified 10 conserved motifs among the SjHSP70 proteins (Fig. 3C, Table S2). While some members, including SjHSP70-02, -06, -10, -12, -13, and -14, contained all 10 motifs, others displayed fewer motifs. The domains in each HSP70 are shown in Fig. 3D. All members possess the conserved HSP70 domain. In addition, SjHSP70-02, -03, -04, -05, -09, and -10 contain a signal peptide.

### Cis-acting elements of *SjHSP70* genes

**Figure 4:**
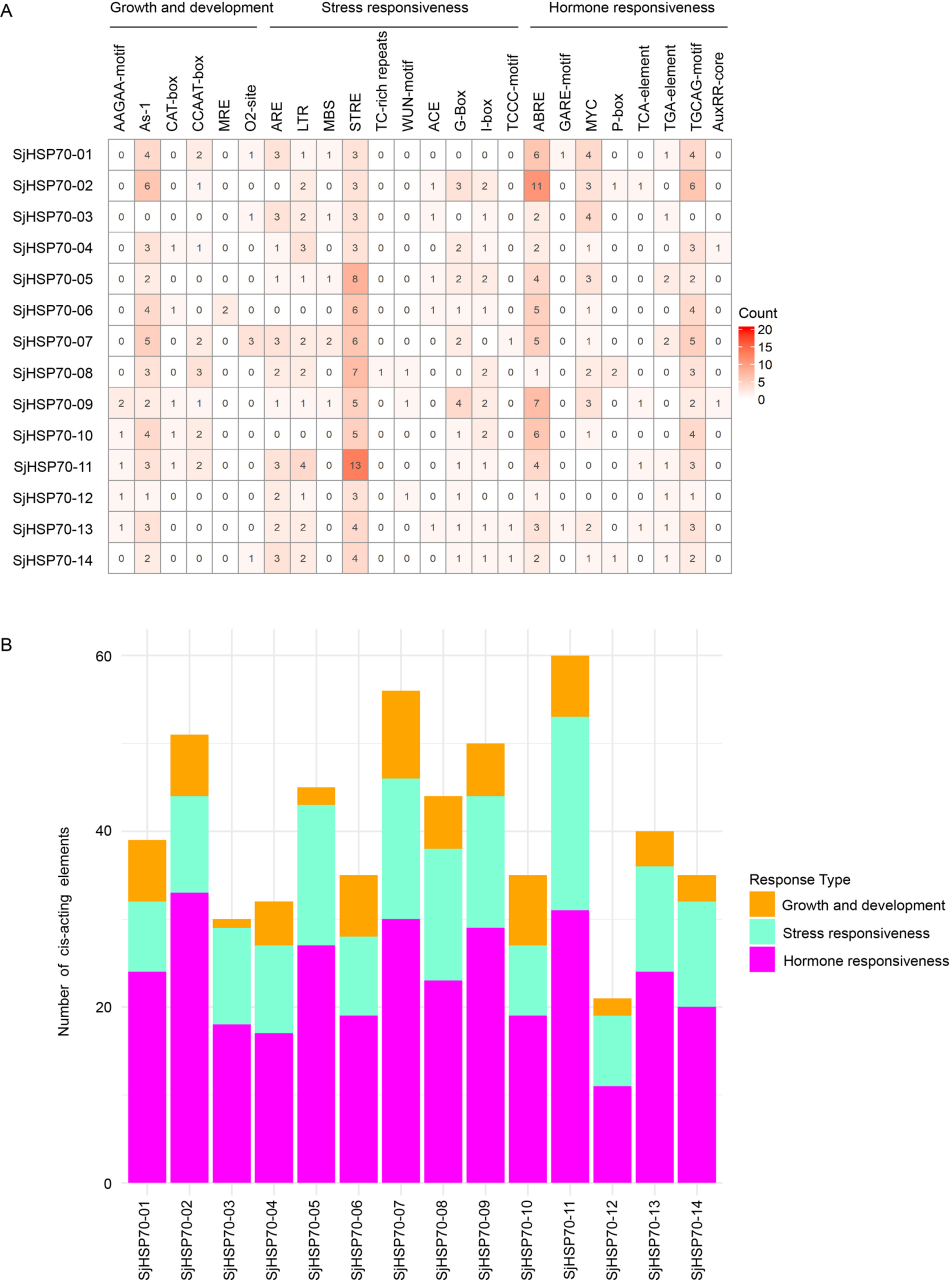
Summary of plant development-, stress inducible-, and phytohormone cis-elements in the promoter regions of *SjHSP70* genes. (A) Number of cis-acting elements in the promoter regions of *SjHSP70* genes. The legend for motifs: AAGAA-motif (related to endosperm expression); As-1 (salicylic acid response); AT-rich (involved in gene expression regulation); CAT-box (meristem expression-related element); CCAAT-box (binds NF-Y transcription factors); MRE (metal response element); O_2_-site (endosperm expression); ARE (hypoxia inducible response); LTR (low-temperature response); MBS (MYB binding site involved in drought response); STRE and TC-rich repeats (defense and stress response); WUN-motif (wound induction response); ACE, G-box, and I-box (light-induced response); TCCC-motif (light-induced responsive element); ABRE (abscisic acid response); GARE-motif, P-box, and MYC (gibberellin-responsive element); TCA-element (salicylic acid response); TGA-element (auxin-response); TGCAG-motif (MeJA-response); AuxRR-core (auxin response). (B) Distribution of cis-elements within each category.

To analyze regulatory elements, the 2.0 kb upstream promoter regions of each *SjHSP70* gene were scanned using PlantCARE. A total of 24 cis-acting elements related to plant development, stress responses, and phytohormone regulation were identified (Fig. 4). All genes contained cis-acting elements related to plant development, except for *SjHSP70-03*, which lacked any development-related elements. In addition, all *SjHSP70s* contained stress response element (STRE) and abscisic acid-responsive element (ABRE). Notably, *SjHSP70-11* had the highest number of STRE (a total of 13), highlighting its potential role in stress response regulation.

### Expression patterns of *SjHSP70* genes across various tissues during different developmental stages and under diverse stress conditions

**Figure 5:**
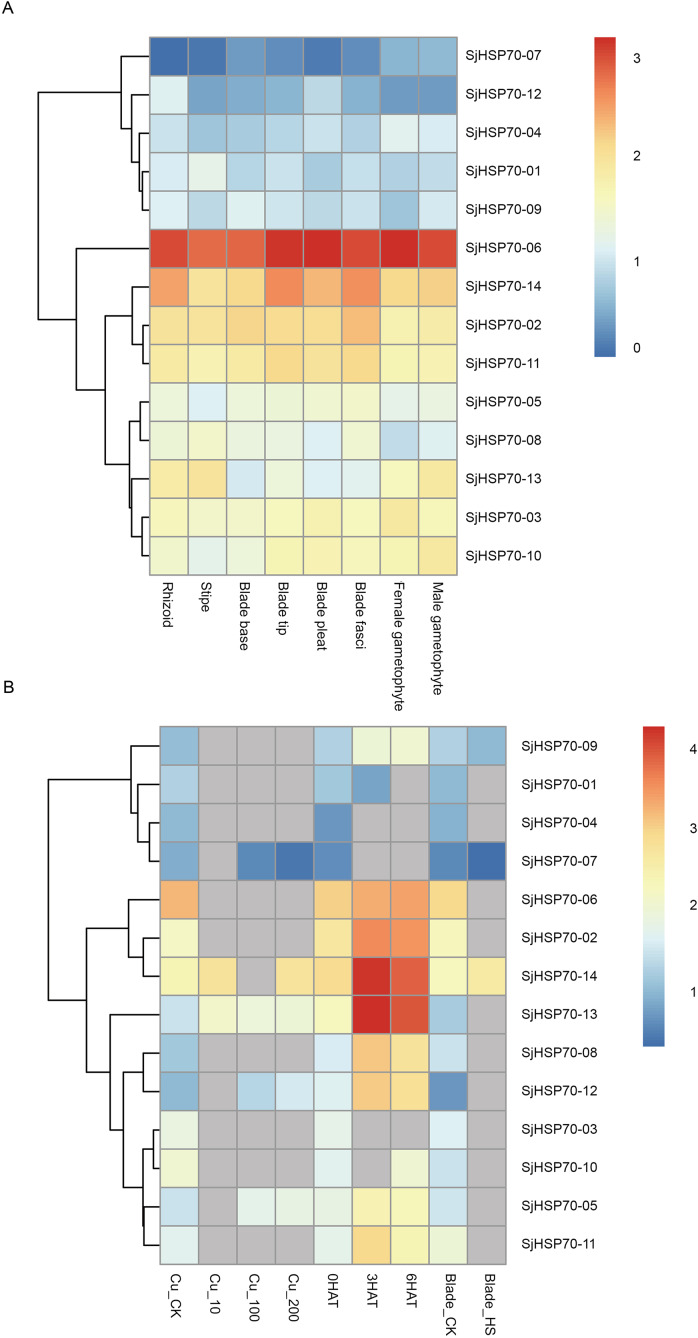
Heatmap of the expression patterns of *SjHSP70*s during different stages in different tissue sites (A) and under different stress treatments (B). Cu_CK: control for Cu^2+^ treatment; Cu_10: 10 μg·L^−1^ Cu^2+^; Cu_100: 100 μg·L^−1^ Cu^2+^; Cu_200: 200 μg·L^−1^ Cu^2+^; 0 HAT: 0 h after treatment at 25 °C; 3 HAT: 3 h after treatment at 25 °C; 6 HAT: 6 h after treatment at 25 °C; Blade_CK: Blade control salinity (30‰); Blade_HS: Blade under hyposaline stress (12‰). The red and blue colors represent higher and lower transcript levels compared to controls, respectively. Gray blocks indicate that the genes were not significant change compared to control.

To examine the expression patterns of the *SjHSP70* genes, transcriptome data from previous studies (Zhang et al., 2019; Chi et al., 2020; Lin et al., 2025) were used for further analyses. Among 14 *SjHSP70* genes examined, only *SjHSP70-07* demonstrated low to almost no expression across various tissues (Fig. 5A, Table S4). The expression profiles of the remaining 13 *SjHSP70s* exhibited notable variations across different tissues. Specifically, *SjHSP70-06* exhibited high expression across all different tissues. *SjHSP70-01*, *-08*, and *-13* had the highest expression levels in stipe compared to other tissues. In contrast, *SjHSP70-10* displayed minimal expression in stipe but high expression in male gametophyte when compared to other tissues. *SjHSP70-04* was predominantly expressed in the female tissue, with relatively lower expression in stipe compared to other tissues. The transcripts of *SjHSP70-03* were most abundant in the female tissue relative to other tissues. *SjHSP70-02*, *-05*, and *-11* showed peak expression levels in the blade fascia tissue compared to other tissues. On the other hand, *SjHSP70-09* and *SjHSP70-12* exhibited the highest expression levels in rhizoids compared to other tissues. These tissue-diverse expression patterns suggest that the SjHSP70 members are potentially involved in distinct biological functions.

**Figure 6:**
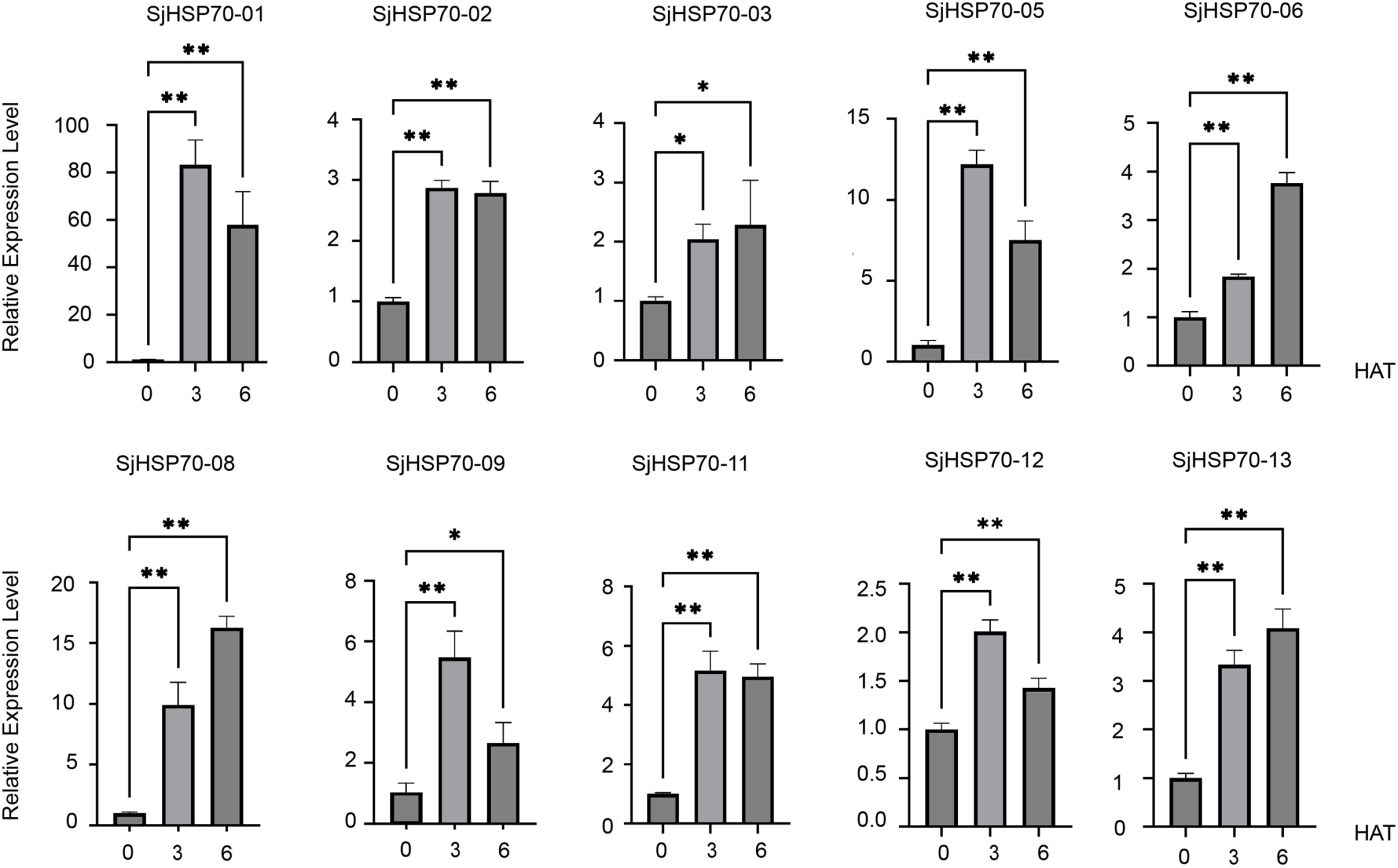
qRT-PCR validation of ten *SjHSP70s* under heat stress. The *β-actin* gene was used as an internal control. All data were obtained from three independent repeats. Error bars represent mean ± SD. HAT: hour after heat treatment at 25 °C; *, *p* < 0.05; **, *p* < 0.01.

The analysis also revealed that most *SjHSP70* genes did not respond significantly to high copper ion or hyposaline stress (Fig. 5B, Table S4). However, the expression of *SjHSP70-13* increased under copper exposure, while *SjHSP70-14* was up-regulated by both high copper ion and hyposaline stress. In addition, most *SjHSP70* genes were up-regulated under heat stress, particularly *SjHSP70-02*, *-05*, *-06*, *-08*, *-09*, *-11*, *-12*, *-13*, and *-14*. Quantitative reverse transcription polymerase chain reaction (qRT-PCR) was performed to validate the RNA-seq results for 10 selected genes under heat stress at 0, 3, and 6 h after treatment (HAT), and the results were consistent with the RNA-seq data (Fig. 6).

### Subcellular location

**Figure 7:**
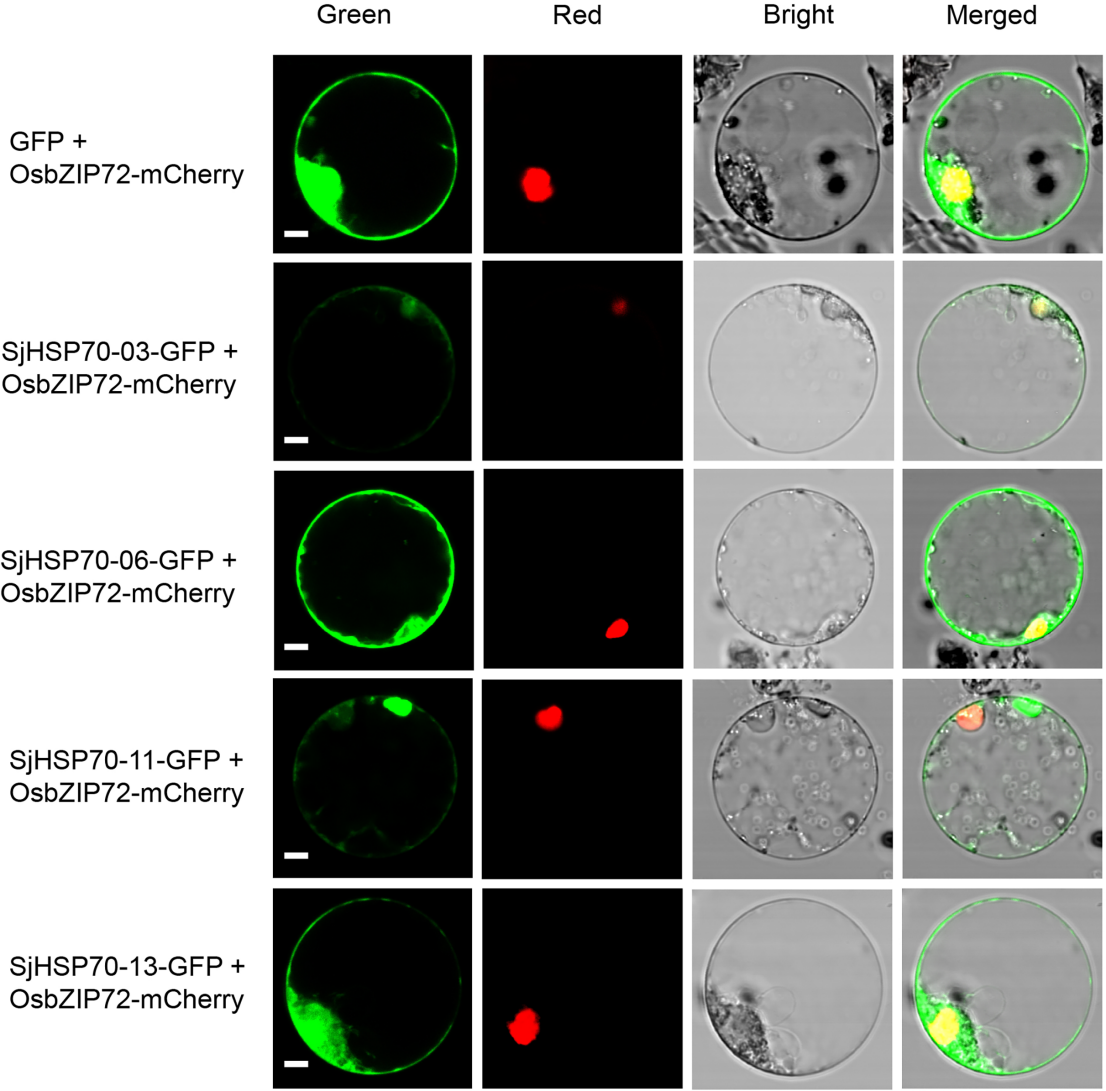
Subcellular localization of four SjHSP70s in rice protoplasts. GFP + OsbZIP72-mCherry: rice protoplast co-transfected with GFP alone and the nucleus marker OsbZIP72-mCherry; SjHSP70-03-GFP + OsbZIP72-mCherry, SjHSP70-06-GFP + OsbZIP72-mCherry, SjHSP70-11-GFP + OsbZIP72-mCherry, and SjHSP70-13-GFP + OsbZIP72-mCherry: rice protoplasts co-transfected with the respective SjHSP70-GFP fusion and OsbZIP72-mCherry; Green, fluorescence under GFP channel; Red, fluorescence under RFP channel; Bright, bright-field image; Merged, merged image of green, red, and bright-field channels. Scale bar, 10 μm.

Four SjHSP70 proteins, namely SjHSP70-03, -06, -11, and -13, were selected for subcellular localization assays. Microscopy revealed that SjHSP70-03, -06, and -13 were mainly localized in the nucleus and cytoplasm, whereas SjHSP70-11 was also detected in the nucleus and cytoplasm but predominantly in an undetermined compartment (Fig. 7). The precise localization of SjHSP70-11 within this compartment requires further investigation.

## Discussions

*S. japonica* is a brown seaweed with high economic value and has been widely cultivated in China. It is an alien species originally introduced from the coasts of the Japan Sea and the Okhotsk Sea (Wang et al., 2018). Over decades of cultivation, new *S. japonica* cultivars have been bred to be more adaptable to high-temperature seawater than populations from its native habitat. Elucidating the molecular mechanisms underlying heat stress responses in *S. japonica* is essential for further breeding improvements. The HSP70 superfamily members, which function as molecular chaperones, play a critical role in cellular defense against various stresses (Usman et al., 2017). In this study, we identified 14 HSP70 family members and analyzed their expression patterns across different tissues, developmental stages, and under multiple abiotic stress conditions.

Phylogenetic analysis grouped 14 HSP70 family members into two subfamilies: 10 as DnaK-type and four as HSP110-type (Fig. 2), consistent with findings in *A. thaliana* and *P. yezoensis* (Lin et al., 2001; Yu et al., 2021). Unlike *HSP70* genes in *A. thaliana* (Lin et al., 2001), *P. yezoensis* (Yu et al., 2021), and *Gracilariopsis lemaneiformis* (Gu et al., 2012), which typically contain one or no introns, *SjHSP70* genes possess multiple introns (Fig. 3A). Similarly, *HSP70* genes in green algae such as *C. reinhardtii*, *Volvox carteri*, and *Ulva prolifera* have six, eight, and five introns, respectively (Cheng et al., 2006; Müller, Igloi & Beck, 1992; Zhang et al., 2012). These results indicate that the exon-intron architectural features of the HSP70 family are more evolutionarily conserved among algal lineages than between algae and terrestrial vascular plants.

Predictive analysis of the promoter regions of *SjHSP70* genes revealed multiple cis-elements associated with development, stress response, and phytohormone response (Fig. 4). A significant number of development-related cis-elements were identified, which correlated with intermediate to relatively high expression levels of all *SjHSP70* genes except *SjHSP70*-*07* across various tissues and developmental stages (Fig. 5A), suggesting that SjHSP70s may play important roles in the development of *S. japonica*. The presence of multiple stress-related cis-elements is also significant. While only a few heat shock elements (HSEs) were found, consistent with previous observations of *HSP70* genes in *P. yezoensis* (Yu et al., 2021), several other stress-responsive elements were present, including low-temperature response elements (LTRs), MYB binding sites (MBSs), STREs, and ABREs. Consistently, 10 out of 14 *SjHSP70* genes (*SjHSP70-02*, *-05*, *-06*, *-08*, *-09*, *-10*, *-11*, *-12*, *-13*, and *-14*) were significantly up-regulated in response to heat stress (Figs. 5B and 6). Collectively, these results strongly support the involvement of SjHSP70s in abiotic stress adaptation, particularly heat tolerance.

Since direct expression systems in *S. japonica* cells remain limited, we employed a rice protoplast transient expression system to determine the subcellular localization of four selected SjHSPs. While phylogenetic analysis clustered SjHSP70-03 and -13 as cytoplasm type, SjHSP70-06 as Cp type, and SjHSP70-11 as Mit type (Fig. 2), all four SjHSP70s were observed to localize to both the nucleus and cytoplasm. Unexpectedly, SjHSP70-11 was also found to predominantly accumulate in an undetermined compartment (Fig. 7). Whether the predictions from phylogenetic analysis are accurate, or whether the heterologous rice cell expression system has inherent limitations in accurately presenting the native subcellular localization of *S. japonica* proteins, requires further investigation.

## Conclusions

In summary, we identified 14 *SjHSP70* genes in *S. japonica*, which were phylogenetically classified into two distinct subfamilies. All encoded SjHSP70s contained the characteristic HSP70 domain. Expression profiling based on publicly available RNA-seq datasets revealed that these genes exhibited diverse expression patterns across different tissues and in response to various environmental stresses. Notably, 10 out of the 14 identified *SjHSP70* genes were up-regulated under heat stress. These results together implicate the potential roles of SjHSP70s in regulating developmental processes and mediating stress responses. Subcellular localization assays in rice protoplasts indicated that SjHSP70-03, SjHSP70-06, and SjHSP70-13 localized to both the nucleus and cytoplasm, whereas SjHSP70-11 was also detected in the nucleus and cytoplasm, but predominantly resided in an undetermined compartment. This study provides a systematic characterization of the *HSP70* gene family in *S. japonica* and offers valuable insights for future research on stress response mechanisms in this species.

## Supplemental Information

10.7717/peerj.21451/supp-1 Supplemental Information 1 Supplemental tables.

10.7717/peerj.21451/supp-2 Supplemental Information 2 Data of qRT-PCR.

10.7717/peerj.21451/supp-3 Supplemental Information 3 MIQE checklist for qRT-PCR.

## Additional Information and Declarations

### Data Availability

The following information was supplied regarding data availability:

The data is available in the Supplemental Files.

Data is available at NCBI, accession numbers PRJNA949272, PRJNA387211, and National Genomics Data Center Data accession numbers PRJCA000815.
